# Practice patterns on the management of secondary hyperparathyroidism in the United States: Results from a modified Delphi panel

**DOI:** 10.1371/journal.pone.0266281

**Published:** 2025-01-31

**Authors:** David E. Henner, Beatrice Drambarean, Teresa M. Gerbeling, Jessica B. Kendrick, William T. Kendrick, Lisa Koester-Wiedemann, Thomas L. Nickolas, Anjay Rastogi, Anis A. Rauf, Brenda Dyson, Michael C. Singer, Pooja Desai, Kathleen M. Fox, Sunfa Cheng, William Goodman

**Affiliations:** 1 Division of Nephrology, Berkshire Medical Center, Pittsfield, MA, United States of America; 2 University of Illinois Hospital & Health Sciences System, Chicago, IL, United States of America; 3 Dialysis Center of Lincoln, Lincoln, NE, United States of America; 4 Division of Renal Diseases and Hypertension, University of Colorado, Aurora, CO, United States of America; 5 Eastern Nephrology Associates, Greenville, NC, United States of America; 6 Division of Nephrology, Washington University School of Medicine, St Louis, MO, United States of America; 7 Division of Nephrology, Columbia University Irving Medical Center, New York, NY, United States of America; 8 Division of Nephrology, UCLA School of Medicine, Los Angeles, CA, United States of America; 9 Nephrology Associates of Northern Illinois, Oakbrook, IL, United States of America; 10 Mississippi, United States of America; 11 Department of Otolaryngology–Head and Neck Surgery, Henry Ford Hospital, Detroit, MI, United States of America; 12 Amgen, Inc., Thousand Oaks, CA, United States of America; Medical College of Wisconsin, UNITED STATES

## Abstract

**Background:**

Secondary hyperparathyroidism (SHPT) is common in patients with chronic kidney disease (CKD). Many recommendations in the Kidney Disease Improving Global Outcomes (KDIGO) CKD-mineral and bone disorder guidelines are supported by modest evidence and predate the approval of newer agents. Therefore, an expert panel defined consensus SHPT practice patterns in the United States with real-world context from the nephrology community.

**Methods:**

Ten US healthcare providers and one patient participated in a modified Delphi method comprising three phases. Consensus was determined via iterative responses to a questionnaire based on the 2009 and 2017 KDIGO guidelines and published literature on the identification, evaluation, monitoring, and interventional strategies for patients with SHPT. The threshold for consensus was 66% agreement.

**Results:**

Panelists generally agreed with KDIGO recommendations, with some differences. Consensus was reached on 42/105 (40%), 95/105 (90.5%), and 105/105 (100%) questions after phases 1, 2, and 3, respectively. Panelists unanimously agreed that SHPT treatment is often started late. There was a preference for serum phosphate level <4.6 mg/dL, and consensus to maintain serum calcium levels <9.5 mg/dL. There was unanimous agreement for vitamin D analogues as first-line options in patients not on dialysis with severe, progressive SHPT and unanimous preference for intravenous calcimimetic, etelcalcetide, in appropriate in-center dialysis patients. Factors such as formularies, dialysis center protocols, and insurance were recognized to influence therapeutic strategies.

**Conclusions:**

Expert consensus was reached on SHPT management, further defining therapeutic strategies and medication use and emphasizing need for treatment early. Despite evidence-based treatment preferences supported by clinical experience, factors other than scientific evidence influence decision making, particularly with medications.

## Introduction

Secondary hyperparathyroidism (SHPT) is a common complication of chronic kidney disease (CKD) resulting in considerable morbidity, including fractures, vascular and soft tissue calcifications, and cardiovascular complications [1–3]. SHPT is characterized by elevated levels of parathyroid hormone (PTH) secondary to dysregulated calcium and phosphate metabolism [4]. SHPT worsens as kidney function deteriorates and is highly prevalent in dialysis patients, including 54% of US adults on dialysis [5].

SHPT management focuses on control of PTH, calcium, and phosphate levels. Key therapeutic strategies include lifestyle management (e.g., dietary phosphate control) and pharmacologic interventions such as phosphate binders and calcium and vitamin D supplements [6]. As SHPT progresses, management requires calcitriol/vitamin D analogues or calcimimetics with etelcalcetide, the most recently developed drug for treatment of SHPT in patients with CKD on dialysis [7]. Parathyroidectomy may be considered in severe SHPT unresponsive to medical treatment [8].

The Kidney Disease Improving Global Outcomes (KDIGO) CKD-mineral and bone disorder (CKD-MBD) Guidelines, last updated in 2017, may inform SHPT management; however, many of its recommendations were ungraded or of level 2 suggesting they lacked moderate or strong underlying clinical evidence [9, 10]. Clinical evidence pertaining to SHPT management including the usefulness of calcimimetics and the benefits of more intensive phosphate management strategies continues to accumulate.

The Delphi method is a consensus-building tool that can provide supplementary clinical context, particularly when clinical guidelines lack robust evidence [11–13]. Thus, an expert panel participated in a Delphi process to define current SHPT practice patterns in the US and develop clinically relevant consensus strategies for the management of SHPT. The panel’s SHPT management perspective provides additional clinical context and real-world insights from the US nephrology community.

## Materials and methods

### The modified Delphi process

The Delphi method is a consensus-building tool that begins with open-ended questions that are refined to summary statements after multiple rounds of anonymous feedback [11]. The modified Delphi method uses published literature, guidelines, or other scholarly work to develop predefined questions and allows participants the opportunity to interact to discuss key points in a face-to-face meeting [11].

**Fig 1:**
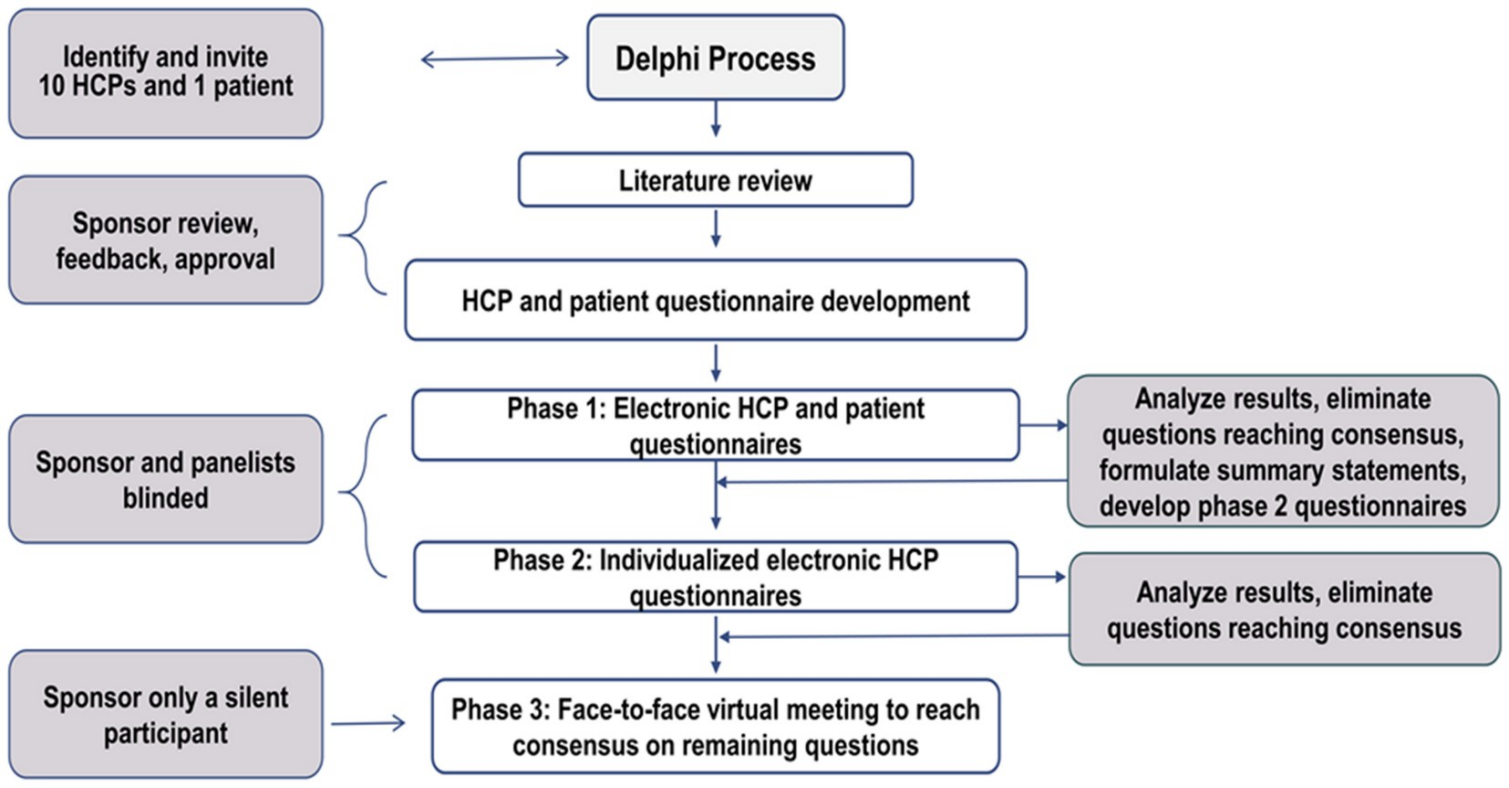
Delphi methodology and study design. HCP, healthcare provider.

In this study, ten healthcare providers (HCPs) and one patient participated in a 3-phase modified Delphi process (**Fig 1**). In phase 1, HCP panelists anonymously completed an electronic questionnaire. The patient completed a separate questionnaire not included in consensus calculations that captured the patient’s perspective. If a panelist did not feel qualified to answer, they were excluded from the consensus calculation for that question. Questions were eliminated from subsequent phases after reaching a prespecified threshold for consensus.

In phase 2, HCP panelists were asked if they would reconsider their response to all questions that did not reach consensus in phase 1 in light of the most common response provided by their anonymous peers. In phase 3, all panelists participated in an unblinded, virtual meeting to briefly discuss statements that had reached consensus in phases 1 and 2, and then discuss the remaining questions until final agreement was reached. The patient provided perspective on key discussion points relating to lifestyle and quality of life factors, side effects of medications, and delays in therapy. The virtual meeting was moderated by an independent facilitator to ensure equal participation. This study was exempt from ethical standards review because no experiment or intervention was conducted. However, written informed consent was obtained from participants.

### Participants

The panelists were US HCPs experienced in the care of patients with SHPT and included six nephrologists [community and academic], one pharmacist, one nurse practitioner, one dietitian, and one surgeon. Expert panelists had relevant knowledge and experience in the management of SHPT and were willing to participate in the entire process. Panelists provided written informed consent and were blinded to the identity of other participants during phases 1 and 2. Contact with the panelists, questionnaire administration, and data acquisition and analyses were conducted by inScience Communications, Springer Healthcare (New York, NY).

### Questionnaire development and administration

Questionnaires were developed based on the KDIGO 2009 and 2017 CKD-MBD guidelines and related literature addressing patient evaluation and monitoring, therapeutic interventions, outcomes, and key patient considerations (e.g., quality of life, involvement in treatment). Questionnaires included a mix of open- and closed-ended questions; there were 126 questions in the HCP questionnaire and 52 in the patient questionnaire (**S1 File**). Twenty-one questions in the HCP questionnaire were qualitative and elicited practice-specific factual information that added context to HCP answers; these were not subject to consensus.

Phase 1 questionnaires were completed from September 28—October 18, 2020, using an online survey platform. The phase 2 individualized questionnaires were completed between October ―October 25, 2020. The phase 3 virtual meeting was held on November 3, 2020.

### Analysis

Quantitative and qualitative data were analyzed using mixed methods. Closed-ended questions were evaluated using descriptive statistics (e.g., percent agreement) throughout all three phases. Open-ended questions in phase 1 were evaluated using thematic analysis to identify key words and phrases to create summary statements for the panelists to agree or disagree with in phases 2 and 3, which were then evaluated using descriptive statistics. The pre-specified consensus threshold was ≥66% agreement among eligible panelists.

## Results

All participants completed all questionnaires. Of the questions requiring consensus, 42/105 (40.0%) reached consensus at the conclusion of phase 1, and 95/105 (90.5%) reached consensus at the conclusion of phase 2. All questions reached agreement by the conclusion of phase 3, with two requiring modification and the addition of one question.

### Evaluation and monitoring

There was agreement with the KDIGO 2017 CKD-MBD guidelines pertaining to the evaluation and monitoring of patients with SHPT (**Table 1**). The participants unanimously agreed that SHPT treatment is often started too late, and it was suggested the course of treatment progresses slower than the disease partly because of the paucity of reliable biomarkers. In agreement, the patient panelist was not prescribed medication for SHPT until their original dialysis initiation, which indicates that SHPT may have gone untreated until CKD stage G5D. When restarting dialysis 27 years later prior to kidney transplant loss, the patient received only vitamin D therapy. The panel recommended serum levels of phosphate, calcium, and PTH to be monitored starting at CKD stage G3a at intervals of every 6 months, 3–6 months at CKD stage G3b, and at least every 3 months at CKD stage ≥G4 (**Table 1**). Panelists agreed that the frequency of monitoring may vary on the basis of rate of disease progression, magnitude of abnormality, or other patient-specific factors.

**Table 1:** Consensus practice patterns for the evaluation, identification, and monitoring of patients with SHPT.

Consideration	Key consensus points (% agreement)	Additional comments and considerations (% agreement)
Timing and frequency of phosphate, calcium, and PTH monitoring (related KDIGO 2017 guidelines: 3.1.1, 3.1.2, and 4.2.1)	Typical monitoring frequency^a^ for CKD stage G3a: 6 months (100); CKD stage G3b: 3–6 months (100); CKD stage G4: 3 months (90); CKD stage G5: 3 months (80–90)^b^; CKD stage G5D: 3 months (90–100)^c^	Begin actively monitoring phosphate, calcium, and PTH at CKD stage G3a with emphasis on serial determination of PTH levels to identify patients with progressively rising or persistently elevated PTH levels in agreement with KDIGO 2017 guidelines 3.1.1 and 4.2.1 (100)
Defining when a patient needs SHPT treatment	Elevated serum PTH (e.g., >300^d^ pg/mL iPTH) for CKD stage G3a and above, often coincident with elevated phosphate levels, typically defines patients requiring treatment for SHPT (100) Serum iPTH ≥1000 pg/mL typically defines severe SHPT^e^ (100)	Rapid progression of SHPT is best determined by evaluating continual/persistent increases with respect to prior measurements rather than specific absolute thresholds (100)

^a^Phosphate, calcium, and PTH are typically measured at the same time. Frequency may vary on the basis of rate of disease progression, magnitude of abnormalities, or other patient-specific factors.

^b^90% agreement for phosphate and calcium, 80% agreement for PTH.

^c^100% agreement for phosphate and calcium, 90% agreement for PTH.

^d^70% agreement to begin treatment for SHPT in a typical patient at serum PTH >300 pg/mL, can vary depending on other factors.

^e^Can vary depending on other factors (e.g., CKD stage).

CKD, chronic kidney disease; iPTH, intact parathyroid hormone; KDIGO, Kidney Disease Improving Global Outcomes; SHPT, secondary hyperparathyroidism.

Biochemical tests were preferred for assessing disease progression (67%), with second-generation intact PTH the most common mode of PTH measurement (80%). Practice setting (e.g., rural vs urban or large dialysis organizations vs non–large dialysis organizations) did not prevent administration of appropriate tests (80%).

Although panelists agreed PTH increases as a percentage of prior levels are a good measure of disease progression, there was consensus that serum PTH levels >300 pg/mL indicate a need for treatment (80%), and dialysis patients are considered out of target at ≥8 times the upper limit of normal for the assay. For a second-generation intact PTH assay with a reference range of 15–65 pg/mL, this would equate to a PTH level ≥520 pg/mL [14].

### Therapeutic interventions

Thresholds for interventions could not be defined in absolute terms for all patients because of patient- and practice-specific factors. Consensus treatment approaches for typical patients with SHPT are summarized in **Table 2**.

**Table 2:** Consensus practice patterns for SHPT interventions.

Intervention	Key consensus points (% agreement)	Additional comments and considerations (% agreement)
Dietary phosphate modification	• No longer sufficient when serum phosphate is >5.5 mg/dL (1.8 mmol/L) (90)	• Preference to target serum phosphate <4.6 mg/dL (1.5 mmol/L) when possible • Difficult to implement, but can be feasible in some patients (70) • Cost and psychological burden are significant factors in success
Phosphate binders	• Initiate^a^: serum phosphate >5.5 mg/dL (1.8 mmol/L) (90) • Evaluate^b^: serum phosphate (89), 1–2 months (80)	• Restrict and avoid calcium- and aluminum-based binders, respectively (80) • Calcium-based binders potentially useful to combat hypocalcemia (100)
Vitamin D/ calcitriol/VDRAs	• Vitamin D analogues (cholecalciferol, ergocalciferol) for first-line PTH-lowering therapy in patients with NDD-CKD unless hypercalcemic (100) • Second-line in DD-CKD; first-line if hypocalcemic (100) • Calcitriol/VDRAs reserved for CKD stages G4–G5 with severe (e.g., PTH >1000 pg/mL [106 pmol/L]) SHPT (80) • Evaluate^b^: 1–2 months (70)	• Useful to combat or prevent hypocalcemia (90) • May cause or exacerbate hypercalcemia • Not viewed as effective for meeting PTH goal as monotherapy for severe SHPT in >25% of patients (78) • May provide benefits to some patients distinct from PTH lowering (90)
Dialysate calcium	• Rarely elevated above 2.5 mEq/L (1.25 μmol/L) and only in specific circumstances (e.g., hypocalcemia after a parathyroidectomy) (100)	• Concerns about vascular calcification and calciphylaxis at concentrations >2.5 mEq/L (1.25 μmol/L)
Calcimimetics	• Cinacalcet: first-line PTH-lowering therapy in patients on home dialysis (100) and second-line for in-center patients on dialysis (90) • Etelcalcetide: first-line PTH-lowering therapy for in-center patients (100) • Initiate^a^: patients with CKD stage G5D (100) with serum PTH >300 pg/mL (31.5 pmol/L) (80), serum phosphate >4.5 mg/dL (80), and serum calcium at or above the normal range (80) • Evaluate^b^: serum PTH (100), 1–2 months (70–90)^c^	• Useful in combating hypercalcemia (90) • Etelcalcetide preferred in appropriate in-center patients (100) • Etelcalcetide used as part of phosphate-lowering strategy (70) • Hypocalcemia and cardiac abnormalities (e.g., arrhythmias) should be considered when initiating calcimimetic therapy (100)
Parathyroidectomy	• For patients with persistently high (e.g., >1000 pg/mL [106 pmol/L]) PTH that is unresponsive to maximum tolerated doses of SHPT medications (100)	• Reduced incidence recently with improved therapeutic options

^a^The point at which the intervention would be started in a typical patient.

^b^The time frame after initiation in which to evaluate efficacy.

^c^Varied by agent; cinacalcet 70%, etelcalcetide 90%.

NDD-CKD, not–dialysis-dependent chronic kidney disease; PTH, parathyroid hormone; SHPT, secondary hyperparathyroidism; VDRA, vitamin D receptor activator.

### Phosphate/phosphorous management

KDIGO 2017 recommendation 4.1.2, based predominantly on epidemiologic evidence linking high phosphate levels with mortality [10], suggests lowering phosphate toward the normal range. The panel agreed (90%) that serum phosphate levels >5.5 mg/dL denote hyperphosphatemia that may not be adequately managed by dietary modification alone, particularly if levels remain >5.5 mg/dL after dietary changes. However, some panelists expressed a preference for a target of <4.6 mg/dL.

All HCPs and the patient agreed that while feasible, barriers such as cost, lifestyle, and other socioeconomic factors limit effective implementation of dietary phosphate restrictions. Thus, phosphate binders are used to control serum phosphate levels. Largely aligning with KDIGO 2017 guideline 4.1.6 restricting calcium-containing binders [10], the panel also suggested avoiding calcium-based binders when possible particularly because of concerns regarding development of vascular calcification in patients with CKD stages G3a–G5D (90%). However, it was recognized that prescription insurance and patient preference considerations often influence the selection of therapeutic strategy (**Table 2**). Common gastrointestinal complications with these agents were also recognized and corroborated by the patient, who noted the large size, poor taste, number of pills, and side effects of constipation or diarrhea.

### Calcium management

While KDIGO 2017 guideline 4.1.3 states that hypercalcemia should be avoided, the lack of a threshold defining hypercalcemia complicates its implementation [15]. Although varying serum calcium reference ranges preclude adoption of universal standards, there was strong consensus to maintain serum calcium levels <9.5 mg/dL, within upper normal range (80%); in patients on dialysis, serum calcium >9.5 mg/dL warrants increased monitoring and consideration of calcium-lowering interventions (100%). In contrast, the need to treat hypercalcemia in asymptomatic cases met only moderate agreement (67%). The consensus hypercalcemia management strategy included decreasing calcium intake (diet and medications; 100%) while stopping or adjusting vitamin D therapy; starting and increasing the dose of calcimimetics were also viewed as reasonable approaches.

Hypocalcemia, a classic characteristic of unmanaged CKD, was agreed upon by the HCPs to start at serum calcium levels <8.2 mg/dL (100%) and was considered severe at levels <7.1 mg/dL (90%), particularly if there are symptoms. The consensus hypocalcemia management strategy included increasing dietary calcium intake, starting or increasing the dose of vitamin D therapies, and/or decreasing the dose of or holding calcimimetics, as needed (100%). Calcitriol and vitamin D analogues may be prioritized to combat or prevent hypocalcemia (90%). Importantly, dialysate calcium concentration >2.5 mEq/L was recommended only in rare circumstances (i.e., hungry bone syndrome) because of vascular calcification concerns at higher concentrations. Serum calcium levels also influence the use of specific therapies to address other laboratory levels such as serum PTH and phosphate, such that hypercalcemia favors the use of calcimimetics while hypocalcemia favors vitamin D therapies for PTH lowering (100%).

### Calcitriol and vitamin D analogues

The panel agreed (90%) that vitamin D is a first-line therapy for PTH management, although this varies based on the severity of CKD and levels of calcium, phosphate, and PTH (**Table 2**). In agreement with KDIGO 2017 recommendation 4.2.2 pertaining to patients with non–dialysis-dependent (NDD)-CKD, the panel recommended calcitriol and vitamin D analogues for patients with CKD stages G4–G5 with severe, progressive SHPT (80%) and recommended considering them first-line options for patients with NDD-CKD and severe, progressive SHPT with or at risk of hypocalcemia (100%); however, in agreement with KDIGO, the panel did not recommend routine use in patients with NDD-CKD. While appropriate first-line therapies, calcitriol and vitamin D analogue monotherapy are ineffective for reaching PTH goals (<25% of patients with severe, progressive SHPT; 78%), although some benefit is seen in ~25% of patients (90%).

When initiating calcitriol or vitamin D analogues, the panel agreed (100%) with KDIGO 2017 recommendation 4.2.2 to begin with low doses and titrate up depending on calcium, phosphate, and PTH levels. When vitamin D therapies are ineffective in managing SHPT, a calcimimetic should be considered for patients on dialysis (90%).

### Calcimimetics

Most panelists indicated using both cinacalcet (once-daily, oral administration) [16] and etelcalcetide (intravenous administration thrice-weekly after hemodialysis) [7] in their practice (90%), with a unanimous consensus strategy to employ them when PTH is significantly elevated or out of goal **(Table 2)**. The panel prioritizes calcimimetics when additional PTH control is needed (e.g., when a patient is refractory to vitamin D therapy) and serum calcium levels are elevated (90%) with careful monitoring and adjustment of concurrent phosphate binders, calcium supplements, or vitamin D therapies. In addition to PTH lowering, 70% of panelists believed that etelcalcetide has uses in phosphate management. Risk of hypocalcemia when starting calcimimetic therapy was a concern for 90% of panelists, and serum calcium levels influenced the initiation (70%), dose (70%), and titration (90%) of calcimimetics.

The panel viewed etelcalcetide as first-line PTH-lowering therapy for in-center dialysis patients (100%) and cinacalcet as first-line therapy in home dialysis patients (100%). Despite these preferences, all panelists believed that factors such as cost, dialysis center protocols, or insurance affect the therapeutic choice or the selection of the appropriate calcimimetic.

### Parathyroidectomy

The panel agreed (100%) that patients with high unresponsive PTH levels despite receiving maximum tolerated doses of medications would be considered refractory to therapy and, thus, candidates for parathyroidectomy. The panelists believed that patients requiring parathyroidectomy present with significant comorbidities, and although quality of life may improve afterward, postoperative complications such as hungry bone syndrome and the pursuant hypocalcemia require continued vigilance.

### Outcomes

In clinical practice, the most common outcomes the panelists attempted to modify with their SHPT treatment paradigm were, in order of priority, bone outcomes (e.g., reducing fractures), serum levels of PTH, serum phosphate, and serum calcium. Mortality as a modifiable outcome had a biphasic distribution with some panelists not ranking it as an important outcome because of the paucity of data demonstrating unequivocal mortality benefits of SHPT treatments. Because SHPT alters skeletal metabolism and is associated with an increased incidence of fractures [17, 18], all panelists agreed that bone turnover and/or bone health should be monitored in patients with SHPT with suitable methods including bone mineral density scans and biochemical assessments such as bone-specific alkaline phosphatase. Interestingly, the patient shared that her “bone health” was managed by her gynecologist and not her nephrologist. While fibroblast growth factor 23 has been implicated in SHPT progression and is an area of increased scientific inquiry [19, 20], it is not currently used in clinical settings (100%).

## Discussion

In this modified Delphi panel study, ten US HCPs reached consensus on SHPT management, including patient identification, evaluation and monitoring, therapeutic strategies, and outcomes. The panel largely agreed with KDIGO recommendations regarding when to initiate monitoring of biochemical factors. However, the panel recommended more frequent monitoring of these laboratory levels by CKD stage than KDIGO recommendations [10]. This reflects a consensus by the panel for greater proactivity in prompt initiation of therapy. The Delphi panel’s recommendations provide additional real-world evidence for management strategies for SHPT that were not well supported or lacked evidence in the KDIGO guidelines. The KDIGO guidelines were last updated in 2017, and new strategies and therapies for managing SHPT have developed since then. Thus, the panel provides the most current strategies and recommendations for providers in the United States.

High levels of phosphate are associated with increased mortality in patients with either NDD-CKD [21] or DD-CKD [3], cardiovascular morbidity, and soft tissue calcifications [22]. The panel echoed KDIGO in that dietary restriction can be difficult to implement and may yield minimal improvement to reach target phosphate levels [10]. Similarly, the panel’s phosphate binder approach aligned with KDIGO 2017 recommendations to avoid aluminum-containing binders and restrict calcium containing binders [10] because increased prevalence of vascular calcification in patients with CKD stages G3a–G5D is associated with increased risk of CVD [23], and calcium-based binders have been linked to increased mortality in dialysis patients compared with calcium-free binders [10, 24]. Also, a recent meta-analysis of clinical trial data showed no advantage and possible harm for utilizing phosphate binders in patients with CKD stage G3b-4, and possible benefit in CKD stage G5D [25]. A key difference in the panel’s phosphate management strategy was the role of calcimimetics, particularly related to the role of etelcalcetide in phosphate lowering. In line with evidence from phase 3 randomized, controlled trials showing decreases in serum phosphate in patients treated with etelcalcetide compared with placebo [26, 27], the consensus was that phosphate lowering may be an added benefit of etelcalcetide in appropriate in-center dialysis patients. Emerging evidence suggests that proactive, intensive phosphate management may have benefits, such as decreased progression of coronary artery calcification [28]. This aligns with the preference of some panelists to target a serum phosphate level <4.6 mg/dL, when possible.

Regarding calcium, the panel agreed with KDIGO to avoid hypercalcemia; however, lack of standardization in serum calcium measurements precluded agreement on strict serum level cutoffs to define hypercalcemia [10, 15]. The panel agreed with recommendation to restrict calcium-containing phosphate binders because of concerns pertaining to vascular calcification and increased mortality with elevated calcium levels. The panel differed slightly from KDIGO on a dialysate calcium concentration of 2.5–3.0 mEq/L and agreed that rarely is it appropriate to raise the concentration >2.5 mEq/L [10].

Calcimimetics lower the activation threshold of the calcium-sensing receptor, enhancing its activity on parathyroid cells and thereby lowering serum PTH [29–32]. The panel’s view of calcimimetics as first-line therapy for lowering PTH in appropriate patients was a notable point of consensus. This consensus was independent of the study sponsor, a manufacturer of calcimimetics, cinacalcet and etelcalcetide. Similar to KDIGO 2017 recommendations, the panel recognized the usefulness of calcitriol and vitamin D analogues in patients not on dialysis; however, they expressed strong preference for calcimimetics in dialysis patients requiring PTH lowering. Although calcitriol and vitamin D analogues are considered first-line options in patients not on dialysis, the panel viewed them as ineffective monotherapies to reach PTH goals in severe cases of SHPT. The panel’s unanimous consensus was to use etelcalcetide as first-line PTH-lowering therapy for in-center dialysis and cinacalcet as first-line therapy for home dialysis. This differs from KDIGO, which does not prioritize PTH-lowering therapies for patients on dialysis [10]. However, despite the panel’s consensus, their real-world experience suggests that factors such as hospital and dialysis center protocols or insurance and payer reimbursement schemes may delay or prevent use of etelcalcetide. For example, in some cases, calcimimetics cannot be used until after a patient has been shown to be refractory to vitamin D therapy. These delays may blunt calcimimetic therapy because earlier initiation (i.e., PTH <600 pg/mL) of etelcalcetide is associated with better control of serum PTH [33]. While cost is another key factor in the use of calcimimetics, the recent inclusion of calcimimetics in the Centers for Medicare and Medicaid Services End Stage Renal Disease (ESRD) Prospective Payment System bundle may impact their US availability [34]. Under this system, providers are reimbursed a fixed amount per patient for resources and procedures related to ESRD care for each dialysis session including, but not limited to, diagnostics testing, medical supplies, and therapeutics [34].

The pathophysiology of SHPT and its complications, for example, bone disease, fractures [10, 18], and cardiovascular abnormalities [17], have been well characterized, and the panel reached consensus for therapeutic strategies to control dysregulated phosphate, calcium, and PTH. However, the impact of modifying these biochemical parameters on hard outcomes, such as mortality, in patients with SHPT remains unknown and requires further investigation. Similarly, current biochemical markers such as PTH are lagging, and new markers allowing earlier identification of patients requiring SHPT treatment are needed.

Identifying and controlling SHPT at early stages (i.e., not waiting until patients start dialysis to begin treatment and ensuring treatment begins in patients with DD-CKD before PTH is significantly elevated [e.g., >600 pg/mL]) was considered an important goal but has many technical and administrative difficulties.

The study has limitations to consider including that the Delphi panel was limited to US providers and patient, thus the recommendations are focused on the management of SHPT in the United States. Management strategies and medication availability for SHPT may differ in other countries. The topic of medication dosage and frequency was not addressed by the panel. The patient viewpoint was limited to one patient; a patient panel of >1 patient was beyond the scope of this project.

## Conclusions

In this study, an expert panel reached consensus on the management of SHPT in the United States. While largely aligned with the KDIGO 2017 CKD-MBD guidelines, the panel expressed key differences based on their clinical experience in SHPT management. This included a preference for proactive patient monitoring to enable earlier intervention, and prioritization of calcimimetics, potentially in combination with calcitriol/vitamin D analogues, as preferred first-line therapy in dialysis patients with SHPT. Etelcalcetide was preferred among approved calcimimetics in appropriate in-center patients. However, costs, dialysis center protocols, and insurance requirements often affect treatment decisions. An area of future endeavor is greater patient education and involvement in the shared decisions for SHPT management. As corroborated by the patient, plain-language, easy to understand educational materials will improve patient comprehension and improve their ability to make appropriate health decisions.

## Supporting information

S1 File (DOCX)

S1 Data (XLSX)

## References

[1] Urena-Torres PA, Vervloet M, Mazzaferro S, Oury F, Brandenburg V, Bover J, et al. Novel insights into parathyroid hormone: Report of the parathyroid day in chronic kidney disease. Clin Kidney J. 2019;12: 269–280. doi: 10.1093/ckj/sfy061 30976408 PMC6452197

[2] Natoli JL, Boer R, Nathanson BH, Miller RM, Chiroli S, Goodman WG, et al. Is there an association between elevated or low serum levels of phosphorus, parathyroid hormone, and calcium and mortality in patients with end stage renal disease? A meta-analysis. BMC Nephrol. 2013;14: 88. doi: 10.1186/1471-2369-14-88 23594621 PMC3658973

[3] Block GA, Klassen PS, Lazarus JM, Ofsthun N, Lowrie EG, Chertow GM. Mineral metabolism, mortality, and morbidity in maintenance hemodialysis. J Am Soc Nephrol. 2004;15: 2208–2218. doi: 10.1097/01.ASN.0000133041.27682.A2 15284307

[4] Cunningham J, Locatelli F, Rodriguez M. Secondary hyperparathyroidism: Pathogenesis, disease progression, and therapeutic options. Clin J Am Soc Nephrol. 2011;6: 913–921. doi: 10.2215/CJN.06040710 21454719

[5] Hedgeman E, Lipworth L, Lowe K, Saran R, Do T, Fryzek J. International burden of chronic kidney disease and secondary hyperparathyroidism: A systematic review of the literature and available data. Int J Nephrol. 2015;2015:184321. doi: 10.1155/2015/184321 25918645 PMC4396737

[6] Bellorin-Font E, Vasquez-Rios G, Martin KJ. Controversies in the management of secondary hyperparathyroidism in chronic kidney disease. Curr Osteoporos Rep. 2019;17: 333–342. doi: 10.1007/s11914-019-00533-x 31485996

[7] Parsabiv [US prescribing information]. Thousand Oaks, CA: Amgen, Inc, 2021.

[8] Apetrii M, Goldsmith D, Nistor I, Siriopol D, Voroneanu L, Scripcariu D, et al. Impact of surgical parathyroidectomy on chronic kidney disease-mineral and bone disorder (ckd-mbd) ‐ a systematic review and meta-analysis. PLoS One. 2017;12: e0187025. doi: 10.1371/journal.pone.0187025 29107998 PMC5673225

[9] Kidney Disease: Improving Global Outcomes (KDIGO) CKD-MBD Work Group. KDIGO clinical practice guideline for the diagnosis, evaluation, prevention, and treatment of chronic kidney disease-mineral and bone disorder (ckd-mbd). Kidney Int Suppl. 2009;113: S1-S130. 10.1038/ki.2009.188 19644521

[10] Kidney Disease: Improving Global Outcomes (KDIGO) CKD-MBD Update Work Group. KDIGO 2017 clinical practice guideline update for the diagnosis, evaluation, prevention, and treatment of chronic kidney disease-mineral and bone disorder (ckd-mbd). Kidney Int Suppl. 2017;7: 1–59. 10.1016/j.kisu.2017.04.001 PMC6340919 30675420

[11] Chalmers J, Armour M. The Delphi technique. In: Handbook of research methods in health social sciences. Singapore: Springer Singapore, 2019; pp.715–735.

[12] Jones J, Hunter D. Consensus methods for medical and health services research. BMJ. 1995;311: 376–380. doi: 10.1136/bmj.311.7001.376 7640549 PMC2550437

[13] Murphy MK, Black NA, Lamping DL, McKee CM, Sanderson CF, Askham J, et al. Consensus development methods, and their use in clinical guideline development. Health Technol Assess. 1998;2: i-iv, 1–88. 9561895

[14] Cavalier E, Delanaye P, Vranken L, Bekaert AC, Carlisi A, Chapelle JP, et al. Interpretation of serum PTH concentrations with different kits in dialysis patients according to the KDIGO guidelines: Importance of the reference (normal) values. Nephrol Dial Transplant. 2011;27: 1950–1956. doi: 10.1093/ndt/gfr535 21940481

[15] Isakova T, Nickolas TL, Denburg M, Yarlagadda S, Weiner DE, Gutierrez OM, et al. KDOQI US commentary on the 2017 KDIGO clinical practice guideline update for the diagnosis, evaluation, prevention, and treatment of chronic kidney disease-mineral and bone disorder (CKD-MBD). Am J Kidney Dis. 2017;70: 737–751. 28941764 10.1053/j.ajkd.2017.07.019

[16] Sensipar [US prescribing information]. Thousand Oaks, CA: Amgen, Inc, 2019.

[17] Muppidi V, Meegada SR, Rehman A: Secondary hyperparathyroidism. In: Statpearls. Treasure Island, FL: StatPearls Publishing; 2021. 32491754

[18] Pimentel A, Ureña-Torres P, Bover J, Luis Fernandez-Martín J, Cohen-Solal M. Bone fragility fractures in CKD patients. Calcif Tissue Int. 2021;108: 539–550. doi: 10.1007/s00223-020-00779-z 33219822 PMC8052229

[19] Bouma-de Krijger A, Vervloet MG. Fibroblast growth factor 23: Are we ready to use it in clinical practice? J Nephrol. 2020;33: 509–527. doi: 10.1007/s40620-020-00715-2 32130720 PMC7220896

[20] Gutierrez OM, Mannstadt M, Isakova T, Rauh-Hain JA, Tamez H, Shah A, et al. Fibroblast growth factor 23 and mortality among patients undergoing hemodialysis. N Engl J Med. 2008; 359: 584–592. doi: 10.1056/NEJMoa0706130 18687639 PMC2890264

[21] Kestenbaum B, Sampson JN, Rudser KD, Patterson DJ, Seliger SL, Young B, et al. Serum phosphate levels and mortality risk among people with chronic kidney disease. J Am Soc Nephrol. 2005;16: 520–528. doi: 10.1681/ASN.2004070602 15615819

[22] Kendrick J, Kestenbaum B, Chonchol M. Phosphate and cardiovascular disease. Adv Chronic Kidney Dis. 2011;18: 113–119. doi: 10.1053/j.ackd.2010.12.003 21406296 PMC4010180

[23] Sørensen IMH, Saurbrey SAK, Hjortkjær H, Brainin P, Carlson N, Ballegaard ELF, et al. Regional distribution and severity of arterial calcification in patients with chronic kidney disease stages 1–5: A cross-sectional study of the Copenhagen chronic kidney disease cohort. BMC Nephrol. 2020;21: 534–544. doi: 10.1186/s12882-020-02192-y 33297991 PMC7726904

[24] Block GA, Raggi P, Bellasi A, Kooienga L, Spiegel DM. Mortality effect of coronary calcification and phosphate binder choice in incident hemodialysis patients. Kidney Int. 2007;71: 438–441. doi: 10.1038/sj.ki.5002059 17200680

[25] Ruospo M, Palmer SC, Natale P, Craig JC, Vecchio M, Elder GJ, et al. Phosphate binders for preventing and treating chronic kidney disease-mineral and bone disorder (CKD-MBD). Cochrane Database Syst Rev. 2018;8: CD006023. doi: 10.1002/14651858.CD006023.pub3 30132304 PMC6513594

[26] Block GA, Bushinsky DA, Cunningham J, Dehmel B, Drueke TB, Ketteler M, et al. Effect of etelcalcetide vs placebo on serum parathyroid hormone in patients receiving hemodialysis with secondary hyperparathyroidism: Two randomized clinical trials. JAMA. 2017;317: 146–155. doi: 10.1001/jama.2016.19456 28097355

[27] Fukagawa M, Yokoyama K, Shigematsu T, Akiba T, Fujii A, Kuramoto T, et al. A phase 3, multicentre, randomized, double-blind, placebo-controlled, parallel-group study to evaluate the efficacy and safety of etelcalcetide (ONO-5163/AMG 416), a novel intravenous calcimimetic, for secondary hyperparathyroidism in Japanese haemodialysis patients. Nephrol Dial Transplant. 2017:32: 1723–1730. 10.1093/ndt/gfw408 PMC5837215 28057872

[28] Isaka Y, Hamano T, Fujii H, Tsujimoto Y, Koiwa F, Sakaguchi Y, et al. Optimal phosphate control related to coronary artery calcification in dialysis patients. J Am Soc Nephrol. 2021;32: 723–735. doi: 10.1681/ASN.2020050598 33547218 PMC7920180

[29] Conigrave AD. The calcium-sensing receptor and the parathyroid: Past, present, future. Front Physiol. 2016;7: 563. doi: 10.3389/fphys.2016.00563 28018229 PMC5156698

[30] Bushinsky DA, Block GA, Martin KJ, Bell G, Huang S, Sun Y, et al. Treatment of secondary hyperparathyroidism: Results of a phase 2 trial evaluating an intravenous peptide agonist of the calcium-sensing receptor. Am J Nephrol. 2015;42: 379–388. doi: 10.1159/000442754 26684933

[31] Goodman WG, Hladik GA, Turner SA, Blaisdell PW, Goodkin DA, Liu W, et al. The calcimimetic agent AMG 073 lowers plasma parathyroid hormone levels in hemodialysis patients with secondary hyperparathyroidism. J Am Soc Nephrol. 2002;13: 1017–1024. doi: 10.1681/ASN.V1341017 11912261

[32] Walter S, Baruch A, Dong J, Tomlinson JE, Alexander ST, Janes J, et al. Pharmacology of AMG 416 (velcalcetide), a novel peptide agonist of the calcium-sensing receptor, for the treatment of secondary hyperparathyroidism in hemodialysis patients. J Pharmacol Exp Ther. 2013;346: 229–240. doi: 10.1124/jpet.113.204834 23674604

[33] Cunningham J, Block GA, Chertow GM, Cooper K, Evenepoel P, Iles J, et al. Etelcalcetide is effective at all levels of severity of secondary hyperparathyroidism in hemodialysis patients. Kidney Int Rep. 2019;4: 987–994. doi: 10.1016/j.ekir.2019.04.010 31317120 PMC6611952

[34] End stage renal disease (ESRD) prospective payment system (PPS). United States Centers for Medicare & Medicaid Services. 2021. Available from: https://www.cms.gov/Medicare/Medicare-Fee-for-Service-Payment/ESRDpayment.

