# Correction: Practice patterns on the management of secondary hyperparathyroidism in the United States: Results from a modified Delphi panel

**DOI:** 10.1371/journal.pone.0323062

**Published:** 2025-04-22

**Authors:** David E. Henner, Beatrice Drambarean, Teresa M. Gerbeling, Jessica B. Kendrick, William T. Kendrick, Lisa Koester-Wiedemann, Thomas L. Nickolas, Anjay Rastogi, Anis A. Rauf, Brenda Dyson, Michael C. Singer, Pooja Desai, Kathleen M. Fox, Sunfa Cheng, William Goodman

After the publication of the article [1] it was noted that the article’s Competing interests statement was incorrect. The Competing interests statement was initially correct in the original submission but due to a misunderstanding and an error during a technical check, part of the statement was removed during an update to the statement. The Competing interests statement is hereby updated to:

“The authors of this manuscript have the following competing interests:

All authors participating in the Delphi study have been compensated for their time and involvement in the study. DEH has served on advisory and speakers’ bureau for Amgen and as consultant for Outset Medical. JBK has served on the advisory board for Fresenius Renal Therapies. WTK has served on speakers’ bureau for Amgen and Akebia Pharmaceuticals. TLN has served on advisory boards and as consultant for Amgen and Pharmacosmos. AAR has served on speakers’ bureau for Amgen and Ardelyx and as consultant for Velphoro. MCS has served as advisor for Medtronic. B Dr, TMG, LK-W, AR, and B Dy have nothing to declare.PD, KMF, SC, and WG are employees of Amgen, Inc.

The competing interest statement does not alter the adherence to PLOS One policies on sharing data and materials. In the interest of scientific sharing, the panellist and patient questionnaires have been provided in the supplemental materials.”

The publisher and authors apologize for the error in the published article.
