# Supplementary material for: Practice patterns on the management of secondary hyperparathyroidism in the United States: Results from a modified Delphi panel
Source: PLoS One. 2025 Jan 31;20(1):e0266281. doi: 10.1371/journal.pone.0266281 (PMC11785329; doi:10.1371/journal.pone.0266281)
Supplement: S1 File — (DOCX) [file pone.0266281.s001.docx]

**Supplementary data**

1. Panelist questionnaire
2. Patient questionnaire

## **1. Panelist Questionnaire**

**Secondary HYERPARATHYROIDISM TREATMENT CONSENSUS**

**DELPHI PANEL – Questionnaire Outline**

**OVERVIEW:**

Secondary Hyperparathyroidism (SHPT) is a common complication of chronic kidney disease (CKD) that results in considerable morbidity and mortality.

The stages of CKD are defined by the glomerular filtration rate (GFR).^1^

| **CKD Stage** | **GFR** | **Description of Kidney Function** |
| --- | --- | --- |
| G1 | ≥ 90 | Normal or High |
| G2 | 60–89 | Mildly Decreased |
| G3a | 45–59 | Mildly to Moderately Decreased |
| G3b | 30–44 | Moderately to Severely Decreased |
| G4 | 15–29 | Severely Decreased |
| G5 | <15 | Kidney Failure |
| G5D | <15 | Kidney Failure with Dialysis |

Treatments for SHPT are mainly geared to control parathyroid hormone (PTH) levels which are influenced by serum levels of calcium, phosphate, and vitamin D. Treatments may include controlling PTH, hyperphosphatemia, hypocalcemia, and vitamin D deficiencies through the use of phosphate binders and dietary control of phosphate intake, calcium supplements, vitamin D supplements, and calcimimetics. Parathyroidectomy is suggested for patients with CKD 3–5D with severe hyperparathyroidism (HPT) who fail to respond to the medical/pharmacologic therapies.

Kidney Disease: Improving Global Outcomes (KDIGO) recommendations in adult patients outlined below include the strength of recommendation as Level 1 (“we recommend”), Level 2 (“we suggest”), or “not graded” (NG), and the quality of the supporting evidence as A (high), B, C, or D (very low) (see chart).^1^


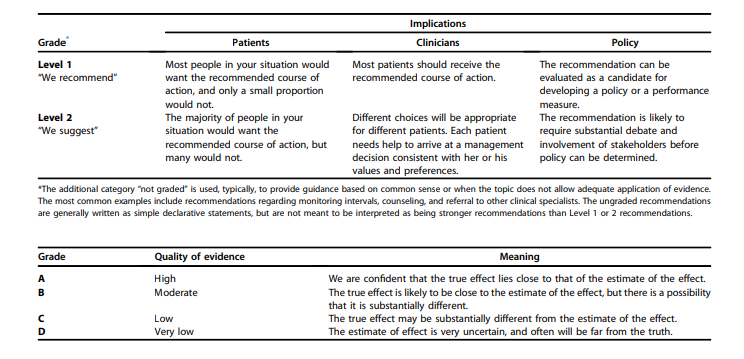
**DELPHI PANEL—Guide to completing the questionnaire**

**Introduction**

Thank you for agreeing to participate in the expert panel. This guide is designed to help you answer the questions in the first round of the Delphi panel.

We recognize that patient care is complex and that treatment plans vary considerably between patients. Therefore, when answering the majority of these questions, please imagine a ‘**typical**’ patient and suggest what you view as a reasonable treatment for that individual. In some cases the questions will focus on patients with specific characteristics and these questions will clearly identify if, in this case, you should NOT answer for the typical patient.

We would like you to provide your opinion based upon **your own experience**. We appreciate that your choices may reflect local formulary restrictions, and in some cases you would not be able to use a product without prior approval/consultation and would be influenced by individual patient factors which may not have been captured in the answer choices. In such cases, please indicate any medications, supplements, or formulations you normally use in your current practice and provide explanations in the space provided.

We recognize that your specialization may involve you in the care of only specific patient types. If you do not feel you can offer an opinion in the management options at any stage, simply note this in the comment section provided.

You are under no obligation to select the use of any specific products. Furthermore, all responses will be anonymized and the sponsor and the other participants will only have access to aggregate data.

Thank you for your participation in this research.

**DELPHI PANEL Questionnaire**

**Evaluation and Monitoring**

**A1. Patient Identification:**

[**KDIGO 2017 4.2.1^1^**](https://kdigo.org/wp-content/uploads/2017/02/2017-KDIGO-CKD-MBD-GL-Update.pdf)

For patients with CKD G3a-G5 not on dialysis, PTH levels should be serially determined starting at stage G3a. KDIGO 2017 changed this from a single value of PTH to serial results for clinical decisions to detect “progressively rising” or “persistently elevated” PTH levels (recommendation strength 2C).

1. To what extent do you agree with this statement? Please mark your answer in the boxes below.

| Strongly agree | Agree | Neutral | Disagree | Strongly disagree |
| --- | --- | --- | --- | --- |
|  |  |  |  |  |

If you disagree or strongly disagree, please type reasons for objection(s) here:

[**KDIGO 2017, 3.1.1^1^**](https://kdigo.org/wp-content/uploads/2017/02/2017-KDIGO-CKD-MBD-GL-Update.pdf)

Serum levels of calcium, phosphate, PTH, and alkaline phosphatase activity should be monitored beginning in CKD G3a (1C).

1. Do you agree with this statement? If you do not agree, at what CKD stage do you begin monitoring these parameters? Please mark your answer in the boxes below and explain.

| Yes | No | The CKD stage (eGFR) at which you begin monitoring | | | | Please explain here |
| --- | --- | --- | --- | --- | --- | --- |
|  |  | G3b  (30–44) | G4  (15–29) | G5  (<15) | G5D  (<15 with dialysis) |  |
|  |  |  |  |  |  |  |

CKD, chronic kidney disease; eGFR, estimated glomerular filtration rate.

[**KDIGO 2017, 3.1.2^1^**](https://kdigo.org/wp-content/uploads/2017/02/2017-KDIGO-CKD-MBD-GL-Update.pdf)

The frequency of monitoring serum calcium, phosphate, and PTH should be based on the presence and magnitude of abnormalities, and the rate of progression of CKD (NG).

1. How often do you monitor these 3 laboratory measures? Please indicate your monitoring frequency for calcium, phosphate, and PTH at each stage of CKD in the boxes below, selecting from the following intervals: every 3 months, every 6 months, every 6–9 months, or yearly.

| CKD stage (eGFR) | Calcium | | | | Phosphate | | | | PTH | | | |
| --- | --- | --- | --- | --- | --- | --- | --- | --- | --- | --- | --- | --- |
|  | Every 3 months | Every 6 months | Every 6–9 months | Yearly | Every 3 months | Every 6 months | Every 6–9 months | Yearly | Every 3 months | Every 6 months | Every 6–9 months | Yearly |
| G3a  (45–59) |  |  |  |  |  |  |  |  |  |  |  |  |
| G3b  (30–44) |  |  |  |  |  |  |  |  |  |  |  |  |
| G4  (15–30) |  |  |  |  |  |  |  |  |  |  |  |  |
| G5  (<15) |  |  |  |  |  |  |  |  |  |  |  |  |
| G5D (<15, on dialysis) |  |  |  |  |  |  |  |  |  |  |  |  |

CKD, chronic kidney disease; eGFR, estimated glomerular filtration rate; PTH, parathyroid hormone.

1. What other parameters or tests do you use to assess disease progression? Please mark your answer in the boxes below by providing details of the assessment tools used.

| Biochemical | Imaging | Clinical | Other (please explain) |
| --- | --- | --- | --- |
|  |  |  |  |

^2^

[**Asian Summit Conference on the implementation of KDIGO 2017**](https://www.sciencedirect.com/science/article/pii/S2468024919314974?via%3Dihub) **3.1.1^2^**

Availability of testing, the eligibility of a patient for testing, and the eligibility of a test to be reimbursed can vary based on the treatment setting (country, type of facility, etc.)

1. How frequently does your practice setting prevent you from conducting tests that you feel are necessary to monitor a patient with secondary hyperparathyroidism? Please mark your answer in the boxes below and explain.

| Very frequently | Somewhat frequently | Infrequently | Very infrequently | Please explain here |
| --- | --- | --- | --- | --- |
|  |  |  |  |  |

[**KDIGO 2017, 4.1.1^1^**](https://kdigo.org/wp-content/uploads/2017/02/2017-KDIGO-CKD-MBD-GL-Update.pdf)

If PTH is progressively rising or persistently above the upper normal limits for the assay, patients should be evaluated (based on serial assessments of phosphate, calcium and PTH levels that are considered together [NG]) for modifiable factors (i.e., hyperphosphatemia, hypocalcemia, vitamin D deficiency).

1. What level of change in PTH over time do you consider to be “rapid progression” necessitating secondary hyperparathyroidism treatment? Please provide as much detail as possible in the box below.
2. At what PTH level do you initiate treatment for severe secondary hyperparathyroidism? Please provide your answer in the box below.
3. How do calcium and phosphate levels influence your answer to questions 6 and 7 (i.e., PTH level or change necessitating treatment)? Please provide as much detail as possible in the box below.
4. In your practice, what are the main characteristics of a patient who needs secondary hyperparathyroidism treatment? Please provide as much detail as possible in the box below.

**A2. Target for Therapy:**

[**KDIGO 2017, 4.2.3^1^**](https://kdigo.org/wp-content/uploads/2017/02/2017-KDIGO-CKD-MBD-GL-Update.pdf)

In patients with CKD G5D, intact PTH levels should be maintained at approximately 2 to 9 times the upper normal limit for the assay; any marked change in either direction should prompt initiation or change in therapy (2C).

1. What is your serum PTH target in a patient who needs secondary hyperparathyroidism treatment? Please mark your answer in the boxes below.

| <150 pg/mL | 150–300 pg/mL | 300–599 pg/mL | 600–1000 pg/mL | ≥1000 pg/mL | Other (please explain here) |
| --- | --- | --- | --- | --- | --- |
|  |  |  |  |  |  |

1. When do you consider your CKD G5D patients to be out of PTH target? Please mark your answer in the boxes below.

| PTH 2–4 x ULN  (130–260 pg/mL iPTH) | PTH 5–7 x ULN  (325–455 pg/mL iPTH) | PTH 8–>9 x ULN  (520–>585 pg/mL iPTH) | Other (please explain here) |
| --- | --- | --- | --- |
|  |  |  |  |

iPTH, intact PTH; PTH, parathyroid hormone; ULN, upper limit of normal.

Values shown are based on an ULN of 65 pg/mL for intact PTH.

1. Do you have a preferred mode of PTH measurement? Please mark your answer in the boxes below.

| Second generation intact PTH | Third generation whole PTH | Other (please explain here) |
| --- | --- | --- |
|  |  |  |

PTH, parathyroid hormone.

[**Position statement by the Italian Society of Nephrology 2020^3^**](https://link.springer.com/article/10.1007/s40620-019-00677-0) **on KDIGO 2017, 4.2.3**In clinical practice, KDIGO 2017 Guideline 4.2.3 does not appear to be followed as the Dialysis Outcomes Practice Patterns Study (DOPPS) data show that secondary hyperparathyroidism treatment is typically started late, such as when PTH has exceeded the target range.[^5^](https://cjasn.asnjournals.org/content/10/1/98.long)

1. Do you agree that treatment of secondary hyperparathyroidism is often started too late? Please mark your answer in the boxes below.

| Agree | Disagree | Please explain here |
| --- | --- | --- |
|  |  |  |

**Interventions**

**B1. Phosphate/Phosphorus Management:**

[**KDIGO 2017, 4.1.5^1^**](https://kdigo.org/wp-content/uploads/2017/02/2017-KDIGO-CKD-MBD-GL-Update.pdf)

In patients with CKD G3a–G5D, decisions about phosphate-lowering treatment should be based on progressively or persistently elevated serum phosphate (NG).

1. What threshold of serum phosphorus levels do you consider hyperphosphatemic, thus a risk for the patient? Please mark your answer in the boxes below and explain.

| >4.5 mg/dL | >5.5 mg/dL | >6 mg/dL | >7 mg/dL | Please explain here |
| --- | --- | --- | --- | --- |
|  |  |  |  |  |

[**KDIGO 2017, 4.1.6, 4.1.7^1^**](https://kdigo.org/wp-content/uploads/2017/02/2017-KDIGO-CKD-MBD-GL-Update.pdf)

- In patients with CKD G3a–G5D receiving phosphate-lowering treatment, the dose of calcium–based phosphate binders should be restricted (2B)
- In patients with CKD G3a–G5D, long-term use of aluminum-containing phosphate binders should be avoided (1C)

1. Do you agree with the above statements? Please mark your answer in the boxes below and explain.

| Agree with both statements | Agree with only the 1^st^ statement | Agree with only the 2^nd^ statement | Disagree with both statements | Please explain here |
| --- | --- | --- | --- | --- |
|  |  |  |  |  |

1. In your experience, what is the optimum period after initiating therapy to confirm efficacy of phosphate binder therapy? Please mark your answer in the boxes below.

| 1–2 months | 3 months | 6 months | Other | Please explain here |
| --- | --- | --- | --- | --- |
|  |  |  |  |  |

[**National Kidney Foundation-Kidney Disease Outcomes Quality Institute** **(NKF-KDOQI) commentary^4^**](https://www.sciencedirect.com/science/article/pii/S0272638617308983?via%3Dihub) **on KDIGO 2017, 4.1.6, 4.1.7**

- Major challenges to a shift away from calcium-containing phosphate binders include potentially higher costs, lower gastrointestinal tolerance based on the patient and type of non-calcium-based phosphate binder used, and unquantified risks with concurrent use of calcimimetics
- Moreover, the evidence regarding the relative efficacy of calcium-containing vs. non-calcium-based phosphate binders on hard clinical outcomes remains limited

1. What is your phosphate binder strategy with your patients with secondary hyperparathyroidism?
2. Is vascular calcification a concern for your patients? Please mark your answer in the boxes below and explain.

| Yes, in all patients with CKD G3a–G5D | Yes, but only in patients with secondary hyperparathyroidism | No | Please explain here |
| --- | --- | --- | --- |
|  |  |  |  |

CKD, chronic kidney disease;

[**KDIGO 2017, 4.1.8^1^**](https://kdigo.org/wp-content/uploads/2017/02/2017-KDIGO-CKD-MBD-GL-Update.pdf)

Dietary phosphate intake should be limited alone or in combination with other treatments (2D).

1. How do you evaluate efficacy of dietary phosphate modification alone? Please explain below and include any lab measures you use.
2. At what stage of phosphorus, calcium, and PTH control is dietary modification alone no longer sufficient for your patients with secondary hyperparathyroidism? Please explain below.

[**NKF-KDOQI commentary^4^**](https://www.sciencedirect.com/science/article/pii/S0272638617308983?via%3Dihub) **on KDIGO 2017, 4.1.8**

Labelling of phosphate content in processed foods is frequently incomplete or inaccurate. Moreover, the consumption of raw, organic, and less processed foods, which typically have lower phosphate content, may be economically prohibitive for many patients as kidney failure disproportionately burdens those living in poverty.

1. How feasible is dietary phosphate restriction in a typical patient with secondary hyperparathyroidism? Please explain below, noting any common barriers to the implementation of this strategy with your patients.

| Very feasible | Somewhat feasible | Not feasible | Please explain here |
| --- | --- | --- | --- |
|  |  |  |  |

**B2. Calcium Management:**

[**KDIGO 2017, 4.1.3^1^**](https://kdigo.org/wp-content/uploads/2017/02/2017-KDIGO-CKD-MBD-GL-Update.pdf)

In patients with CKD G3a–G5D, hypercalcemia should be avoided (2C).

[**NKF-KDOQI commentary^4^**](https://www.sciencedirect.com/science/article/pii/S0272638617308983?via%3Dihub) **on KDIGO 2017, 4.1.3**

The lack of established definitions regarding abnormal calcium levels complicates implementation of the recommendation.

1. At what thresholds do you consider a patient to have mild, moderate, or severe hypercalcemia? Please indicate the thresholds you use in the boxes below and explain.

*Question revised by the panel in phase 3 to read: At what threshold would you be concerned about hypercalcemia in patients on dialysis with SHPT?*

| Mild hypercalcemia | Moderate hypercalcemia | Severe hypercalcemia | Please explain here |
| --- | --- | --- | --- |
|  |  |  |  |

1. What is the calcium level threshold that you attempt to stay below in patients with secondary hyperparathyroidism? Please indicate the thresholds you use in the boxes below and explain.

| 8.5 mg/dL | 9.5 mg/dL | 10.5 mg/dL | Other | Please explain here |
| --- | --- | --- | --- | --- |
|  |  |  |  |  |

[**KDIGO 2017, 4.1.4^1^**](https://kdigo.org/wp-content/uploads/2017/02/2017-KDIGO-CKD-MBD-GL-Update.pdf)

In patients with CKD G5D, a dialysate calcium concentration between 1.25 and 1.50 mmol/L (2.50–3.00 mEq/L) is suggested.

[**NKF-KDOQI commentary^4^**](https://www.sciencedirect.com/science/article/pii/S0272638617308983?via%3Dihub) **on KDIGO 2017, 4.1.3**

Guidance to avoid hypercalcemia may prompt greater use of interventions aimed at reducing calcium levels, such as low dialysate calcium concentration. KDOQI cautions against this as low dialysate calcium has been linked to increased risk of arrhythmia and heart failure in observational studies.

1. What is your typical practice pattern regarding the dialysate calcium concentration for patients with secondary hyperparathyroidism? Please mark your answer in the boxes below and explain.

| Maintain < 2.5 mEq/L | Maintain > 2.5 mEq/L | Other | Please explain here |
| --- | --- | --- | --- |
|  |  |  |  |

1. Do you raise the dialysate calcium concentration above 2.5 mEq/L for your patients with secondary hyperparathyroidism? Please mark your answer in the boxes below and explain.

| Yes, always | Yes, sometimes | No, never | Please explain here |
| --- | --- | --- | --- |
|  |  |  |  |

1. How do you manage hypercalcemia in your patients with secondary hyperparathyroidism? Please explain in the box below, providing as much detail as possible.
2. Do you treat asymptomatic hypercalcemia in patients with secondary hyperparathyroidism? Please mark your answer in the boxes below and explain.

| Yes, always | Yes, sometimes | No, never | Please explain here |
| --- | --- | --- | --- |
|  |  |  |  |

[**KDIGO 2017, 4.1.3^1^**](https://kdigo.org/wp-content/uploads/2017/02/2017-KDIGO-CKD-MBD-GL-Update.pdf)

Correction of hypocalcemia should be individualized rather than implemented for all patients.

[**NKF-KDOQI commentary^4^**](https://www.sciencedirect.com/science/article/pii/S0272638617308983?via%3Dihub) **on KDIGO 2017, 4.1.3**

The lack of established definitions regarding abnormal calcium levels complicates implementation of the recommendation.

1. At what thresholds do you consider a patient to have mild, moderate, or severe hypocalcemia? Please indicate the thresholds you use in the boxes below and explain.

| Mild hypocalcemia | Moderate hypocalcemia | Severe hypocalcemia | Please explain here |
| --- | --- | --- | --- |
|  |  |  |  |

[**NFK-KDOQI commentary^4^**](https://www.sciencedirect.com/science/article/pii/S0272638617308983?via%3Dihub) **and** [**Position of Italian Society of Nephrologists^3^**](https://link.springer.com/article/10.1007/s40620-019-00677-0) **on KDIGO 2017, 4.1.3**

It is plausible that lower levels of serum calcium can be tolerated if they are asymptomatic.

1. How do you manage hypocalcemia, including asymptomatic cases, in your patients with secondary hyperparathyroidism? Please explain in the box below, providing as much detail as possible.
2. What is the role of calcitriol or vitamin D analogues and/or calcium supplements in your management of blood calcium levels in patients with secondary hyperparathyroidism? Please provide your answer in the box below.

[**KDIGO 2017, 4.1.3, rationale^1^**](https://kdigo.org/wp-content/uploads/2017/02/2017-KDIGO-CKD-MBD-GL-Update.pdf)

The prevalence of hypocalcemia may have increased after the introduction of calcimimetics into the clinic.

[**NKF-KDOQI commentary^4^**](https://www.sciencedirect.com/science/article/pii/S0272638617308983?via%3Dihub) **on KDIGO 2017, 4.1.6, 4.1.7**

Calcimimetic-induced hypocalcemia may be accompanied by greater use of calcium-containing phosphate binders.

1. In general, how concerned are you about hypocalcemia in patients with secondary hyperparathyroidism on dialysis when starting a calcimimetic? Please mark your answer in the boxes below.

| Very concerned | Somewhat concerned | Not concerned | Please explain here |
| --- | --- | --- | --- |
|  |  |  |  |

1. Do serum calcium levels influence calcimimetic treatment in terms of type, dose, titration, etc.? Please describe in detail in each box below.

| Type of calcimimetic | Initiation | Dosage | Titration | Other considerations |
| --- | --- | --- | --- | --- |
|  |  |  |  |  |

**B3. Calcitriol/Vitamin D Analogue Management:**

[**KDIGO 2017, 4.2.2^1^**](https://kdigo.org/wp-content/uploads/2017/02/2017-KDIGO-CKD-MBD-GL-Update.pdf)

- - In patients not on dialysis, calcitriol and vitamin D analogues should not be routinely used in patients with CKD G3a-G5 (2C)
  - Calcitriol and vitamin D analogues should be reserved for patients with CKD G4-G5 with severe and progressive HPT (NG)

1. Do you agree with the above statements? Please mark your answer in the boxes below and explain.

| Agree with both statements | Agree with only the 1^st^ statement | Agree with only the 2^nd^ statement | Disagree with both statements | Please explain here |
| --- | --- | --- | --- | --- |
|  |  |  |  |  |

1. What is your vitamin D strategy in patients with secondary hyperparathyroidism? Please provide your answer in the box below.

[**Position of Italian Society of Nephrologists^3^**](https://link.springer.com/article/10.1007/s40620-019-00677-0) **on KDIGO 2017, 4.1.3**

Calcimimetic, rather than calcium or vitamin D, dose adjustment may be the best course of action in symptomatic hypocalcemia.

1. What therapeutic agent do you adjust first in patients with symptomatic hypocalcemia? Please mark your answer in the box below and provide the rationale for your selection.

| Calcimimetics | Calcitriol/Vitamin D analogues | Calcium | Other | Please explain here |
| --- | --- | --- | --- | --- |
|  |  |  |  |  |

[**NKF-KDOIQ commentary^4^**](https://www.sciencedirect.com/science/article/pii/S0272638617308983?via%3Dihub) **on KDIGO 2017, 4.2.2**

A potential unintended consequence of the recommendation to reserve calcitriol or vitamin D analogues for stages CKD G4−G5 with severe and progressive secondary hyperparathyroidism–that is undefined by KDIGO–may be decreased attention to disordered mineral metabolism and PTH levels over time, resulting in less aggressive management of secondary hyperparathyroidism, which can lead to progression to tertiary hyperparathyroidism, necessitating parathyroidectomy or the use of costlier medications.

1. How do you define severe secondary hyperparathyroidism? Please provide your answer in the box below, providing as much detail as possible.
2. Has your management strategy for severe and progressive secondary hyperparathyroidism changed as a result of the 2017 KDIGO guidelines? Please mark your answer in the boxes below and explain.

| Yes | No | If yes, please explain here |
| --- | --- | --- |
|  |  |  |

1. Have the 2017 KDIGO guidelines changed the frequency at which you monitor mineral metabolism and PTH levels in patients with CKD in your practice? Please mark your answer in the box below and explain.

| Yes, more frequently | Yes, less frequently | No | Please explain here |
| --- | --- | --- | --- |
|  |  |  |  |

[**KDIGO 2017, 4.2.2^1^**](https://kdigo.org/wp-content/uploads/2017/02/2017-KDIGO-CKD-MBD-GL-Update.pdf)

If initiated for severe and progressive SHPT, calcitriol or vitamin D analogues should be started with low doses, independent of the initial PTH concentration, and then titrated based on the PTH response.

1. What is your clinical rationale for treating severe and progressive secondary hyperparathyroidism with calcitriol or vitamin D analogues and when do you prioritize their use over other treatments? Please provide your answer in the box below.
2. What are your considerations for the starting dose of vitamin D analogues and your titration strategy for severe and progressive secondary hyperparathyroidism? Please provide your answer in the box below.
3. In your experience, what proportion of patients with severe, progressive secondary hyperparathyroidism benefit from treatment with calcitriol and vitamin D analogues? Please mark your answer in the boxes below. *Revised into 2 questions by the panel in phase 3. Q41A: What proportion of patients with severe, progressive SHPT reach PTH goal from calcitriol/VDRAs? Q41B: What proportion of patients achieves any benefit from calcitriol/VDRAs despite severe, progressive SHPT?*

| Beneficial to almost all | Beneficial to at least 50% | Beneficial for a few | Never beneficial |
| --- | --- | --- | --- |
|  |  |  |  |

1. Are there patient subtypes or characteristics (please consider ALL patients, not just “typical” patients) that would call for a first-line therapy other than calcitriol or vitamin D analogues? Please mark your answer in the boxes below and explain.

| Yes | No | Please explain here |
| --- | --- | --- |
|  |  |  |

1. In your experience, what is the optimum period after initiating therapy to confirm efficacy of vitamin D therapy in patients with secondary hyperparathyroidism? Please mark your answer in the boxes below.

| 1-2 months | 3 months | 6 months | Other | Please explain here |
| --- | --- | --- | --- | --- |
|  |  |  |  |  |

1. What is your course of action when vitamin D therapy is ineffective in managing secondary hyperparathyroidism? Please mark your answer in the box below.

**B4. PTH Management:**

[**KDIGO 2017, 4.2.4^1^**](https://kdigo.org/wp-content/uploads/2017/02/2017-KDIGO-CKD-MBD-GL-Update.pdf)

In patients with CKD G5D requiring PTH-lowering therapy, calcimimetics, calcitriol, or vitamin D analogues, or a combination of calcimimetics with calcitriol or vitamin D analogues are recommended (2B).

1. What is your calcimimetic strategy in patients with secondary hyperparathyroidism? Please provide your answer in the box below.
2. At what stage of secondary hyperparathyroidism disease severity do you consider adding a calcimimetic? Please explain in the box below.
3. KDIGO recommendations for PTH-lowering therapies are not prioritized. Do you prioritize treatment choices or do you have a preferred sequence of therapy? Please mark your answer in the boxes below and explain.

| Yes | No | Please explain here |
| --- | --- | --- |
|  |  |  |

1. When do you prioritize calcimimetics as the treatment for secondary hyperparathyroidism? Please write your answer in the box below.
2. Are there patient subtypes or characteristics (please consider ALL patients, not just “typical” patients) that would call for a first-line PTH-lowering therapy other than calcimimetics? Please mark your answer in the boxes below and explain.

| Yes | No | Please explain here |
| --- | --- | --- |
|  |  |  |

1. Are there factors other than scientific evidence that impact your initial therapeutic choice for patients with CKD G5D requiring PTH-lowering therapy? Please mark your answer in the boxes below and explain.

| Yes | No | Please explain here |
| --- | --- | --- |
|  |  |  |

[**NFK-KDOQI commentary^4^**](https://www.sciencedirect.com/science/article/pii/S0272638617308983?via%3Dihub) **on KDIGO 2017, 4.2.4**

The selection of medication from those listed in KDIGO 4.2.4 should account for concomitant medications as well as serum calcium and phosphate levels of the individual patient.

1. What concomitant medications are of concern when initiating treatment of secondary hyperparathyroidism? Please list and explain in the box below.

[**Position statement by the Italian Society of Nephrology 2020^3^**](https://link.springer.com/article/10.1007/s40620-019-00677-0) **KDIGO 2017, 4.2.4**

- Several studies show superior efficacy of cinacalcet as compared with active vitamin D or its analogues for control of PTH levels as well as other mineral metabolism markers (e.g., serum calcium, serum phosphate, and fibroblast growth factor 23 [FGF23]). Thus, calcimimetics should be seen as first line therapy to control secondary hyperparathyroidism in patients with moderately-to-severely elevated serum calcium levels and elevated serum phosphate levels
- Active vitamin D or its analogues should be the first choice of therapy to control secondary hyperparathyroidism in patients with hypocalcemia

1. Do you agree with these statements? Please mark your answer in the boxes below and explain:

| Agree with both statements | Agree with only the 1^st^ statement | Agree with only the 2^nd^ statement | Disagree with both statements | Please explain here |
| --- | --- | --- | --- | --- |
|  |  |  |  |  |

- [**KDIGO 2017**](https://kdigo.org/wp-content/uploads/2017/02/2017-KDIGO-CKD-MBD-GL-Update.pdf) guidelines recognized the clinical attributes of IV calcimimetics^1^; however, these were not considered in their recommendations which predate the clinical approval of etelcalcetide
- In the time since the publication of the KDIGO 2017 guidelines, multiple phase 3 trials[^6^](https://jamanetwork.com/journals/jama/fullarticle/2596294)^,^[^7^](https://jamanetwork.com/journals/jama/fullarticle/2596293)^,^[^8^](https://academic.oup.com/ndt/article/32/10/1723/2838374) have demonstrated that etelcalcetide, an IV calcimimetic administered 3 times weekly after hemodialysis, is both superior to placebo^7,8^ and non-inferior to cinacalcet^6^ in reducing PTH levels in adult patients with CKD on hemodialysis. Etelcalcetide has been approved by the US Food and Drug Administration.

1. In your practice, do you use cinacalcet or etelcalcetide for secondary hyperparathyroidism? Please mark your answer in the boxes below and explain.

| Yes, both | Yes, only cinacalcet | Yes, only etelcalcetide | No, neither | Please explain here |
| --- | --- | --- | --- | --- |
|  |  |  |  |  |

1. What clinical parameters and biomarkers do you use to identify patients who are appropriate for cinacalcet or etelcalcetide therapy? Please mark your answers, choosing all that apply, in the boxes below and explain, being as specific as possible.

| Parameter/  Biomarker | Drug | Status/Threshold | | | | | | | | | | | | | | Please explain here |
| --- | --- | --- | --- | --- | --- | --- | --- | --- | --- | --- | --- | --- | --- | --- | --- | --- |
| Serum phosphate (mg/dL) |  | >4.5 | | >5.5 | | | >6 | | | >7 | | Other | | | |  |
|  | Cinacalcet |  | |  | | |  | | |  | |  | | | |  |
|  | Etelcalcetide |  | |  | | |  | | |  | |  | | | |  |
| PTH level  (pg/mL) |  | <150 | 150-299 | | | 300–599 | | | | 600–1000 | | | | ≥1000 | |  |
|  | Cinacalcet |  |  | | |  | | | |  | | | |  | |  |
|  | Etelcalcetide |  |  | | |  | | | |  | | | |  | |  |
| CKD stage |  | G3a | | G3b | | | | G4 | | | G5 | | | | G5D |  |
|  | Cinacalcet |  | |  | | | |  | | |  | | | |  |  |
|  | Etelcalcetide |  | |  | | | |  | | |  | | | |  |  |
| Serum calcium (mg/ dL) |  | <LLN | | | normal range | | | | >ULN | | | | Other | | |  |
|  | Cinacalcet |  | | |  | | | |  | | | |  | | |  |
|  | Etelcalcetide |  | | |  | | | |  | | | |  | | |  |
| Comorbidities | Cinacalcet |  | | | | | | | | | | | | | |  |
|  | Etelcalcetide |  | | | | | | | | | | | | | |  |
| Concomitant medications | Cinacalcet |  | | | | | | | | | | | | | |  |
|  | Etelcalcetide |  | | | | | | | | | | | | | |  |
| Other | Cinacalcet |  | | | | | | | | | | | | | |  |
|  | Etelcalcetide |  | | | | | | | | | | | | | |  |

CKD, chronic kidney disease; LLN, lower limit of normal; PTH, parathyroid hormone; ULN, upper limit of normal.

1. If you use calcimimetics, which of the following best describes the place in therapy of cinacalcet and etelcalcetide for patients with secondary hyperparathyroidism? Please mark your answer in the boxes below and explain. *Question revised into 3 parts by the panel in phase 3.* *Q55A: Which best describes the place in therapy of cinacalcet in patients with SHPT on home dialysis? Q55B: Which best describes the place in therapy of cinacalcet in patients with SHPT on in-center dialysis? Q55B: Which best describes the place in therapy of etelcalcetide in patients with SHPT on in-center dialysis?*

| Drug | Place in therapy | | | Please explain here |
| --- | --- | --- | --- | --- |
|  | First-line | Second-line | Third-line |  |
| Cinacalcet |  |  |  |  |
| Etelcalcetide |  |  |  |  |

*New question added by the panel in phase 3: Which calcimimetic do you prefer in patients with SHPT on in-center dialysis?*

1. If you use etelcalcetide, do you prescribe it more or less frequently than cinacalcet? Please mark your answer in the boxes below and explain why.

| More frequently | Equally frequently | Less frequently | Please explain here |
| --- | --- | --- | --- |
|  |  |  |  |

1. Does etelcalcetide have a role in your phosphate lowering strategy? Please explain.
2. How do you evaluate the efficacy of etelcalcetide in patients with secondary hyperparathyroidism? Please explain below and include any lab measures you use.
3. In your experience, what is the optimum period after initiating therapy to confirm efficacy of cinacalcet or etelcalcetide therapy in patients with secondary hyperparathyroidism? Please mark your answer in one of the boxes below.

| Drug | Optimum period to assess efficacy | | | | Please explain here |
| --- | --- | --- | --- | --- | --- |
|  | 1–2 months | 3 months | 6 months | Other |  |
| Cinacalcet |  |  |  |  |  |
| Etelcalcetide |  |  |  |  |  |

**B5. Parathyroidectomy:**

[**KDIGO 2017, 4.2.5^1^**](https://kdigo.org/wp-content/uploads/2017/02/2017-KDIGO-CKD-MBD-GL-Update.pdf)

In patients with CKD G3a–G5D with severe HPT who fail to respond to medical or pharmacological therapy, parathyroidectomy is recommended (2B).

1. What clinical criteria do you use to define treatment failure in severe HPT and at what point do you consider a patient to be refractory to medical/pharmacological therapy and an appropriate candidate for parathyroidectomy?

**Outcomes/biomarkers**

**C1. Fibroblast Growth Factor 23**

The levels of FGF23 and have been associated with progression of CKD, development of secondary hyperparathyroidism, and poor clinical outcomes.[^9^](https://www.nejm.org/doi/10.1056/NEJMoa0706130?url_ver=Z39.88-2003&rfr_id=ori:rid:crossref.org&rfr_dat=cr_pub%20%200www.ncbi.nlm.nih.gov)^,^[^10^](https://link.springer.com/article/10.1007%2Fs40620-020-00715-2)

1. Do you monitor levels of FGF23 in your practice? Please mark your answer in the boxes below and explain why. If yes, please describe the assay you use.

| Yes | No | Please explain here & provide the assay used |
| --- | --- | --- |
|  |  |  |

**C2. Bone Turnover**

1. Do you monitor bone turnover or bone health in your patients with secondary hyperparathyroidism? Please mark any tests performed or markers that you use.

| Test/Marker | Mark if used | Please explain here |
| --- | --- | --- |
| Bone Mineral Density (BMD) test (e.g., DXA scan) |  |  |
| Bone-specific alkaline phosphatase (BAP) |  |  |
| Osteocalcin |  |  |
| iPTH |  |  |
| Pro collagen type I N-terminal propeptide |  |  |
| Urine N-terminal telopeptide of type I collagen |  |  |
| C-terminal telopeptide of type I collagen |  |  |
| Other |  |  |

DXA, dual-energy x-ray absorptiometry; iPTH intact PTH.

**C3. Outcomes of Greatest Interest**

1. Please list the 5 most important clinical outcomes that you try to modify with your treatment paradigm in your patients with secondary hyperparathyroidism. Please list the outcomes from 1 (most important) to 5 (least important).

| 1 (most important) | 2 | 3 | 4 | 5 (least important) |
| --- | --- | --- | --- | --- |
|  |  |  |  |  |

**Patient Focus**

1. How receptive are your patients to increased monitoring of secondary hyperparathyroidism-related biochemical parameters? Please mark your answer in the box below.

| Very receptive | Receptive | Unreceptive | Very unreceptive |
| --- | --- | --- | --- |
|  |  |  |  |

What factors make your patients more or less receptive to increased monitoring of secondary hyperparathyroidism? Please provide your answers in the boxes below.

| 1. More receptive to increased monitoring | 1. Less receptive to increased monitoring |
| --- | --- |
|  |  |

1. How aware is the typical patient of the diversity of available treatment options for secondary hyperparathyroidism? Please mark your answer in the boxes below.

| Very aware | Somewhat aware | Unaware | Not sure |
| --- | --- | --- | --- |
|  |  |  |  |

1. How involved are patients in the selection of therapy for managing secondary hyperparathyroidism? Please mark your answer in the boxes below.

| Very involved | Somewhat involved | Minimally involved | Not involved |
| --- | --- | --- | --- |
|  |  |  |  |

1. In what percentage of your patients is cost likely to factor into the therapeutic choices made by your patients with secondary hyperparathyroidism? Please mark your answer in the box below.

| >75% | 50–75% | 25–49% | <25% | Please explain here |
| --- | --- | --- | --- | --- |
|  |  |  |  |  |

1. How do you think your patients with secondary hyperparathyroidism rank the following factors when deciding on treatment options? Please mark your answers 1–6 below, with 1 indicating the most important factor and 5 indicating the least important factor.

| Convenience | Cost borne by patient | Physician opinion | Pill burden^a^ | Scientific evidence | Other (please specify) |
| --- | --- | --- | --- | --- | --- |
|  |  |  |  |  |  |

^a^Pill burden is the total number of oral medications taken per day.

Adherence to therapy may correlate with improved clinical outcomes (e.g., adhering to cinacalcet, an oral calcimimetic, may reduce hospitalizations of patients with secondary hyperparathyroidism [^11^](https://www.drugsincontext.com/cinacalcet-adherence-in-dialysis-patients-with-secondary-hyperparathyroidism-in-lombardy-region-clinical-implications-and-costs/))

1. In your experience, what percentage of patients with secondary hyperparathyroidism refuses a therapy because of pill burden (total number of oral medications taken per day)?

| >75% | 50–75% | 25–49% | <25% | Please explain here |
| --- | --- | --- | --- | --- |
|  |  |  |  |  |

1. In your experience, what factors are most commonly associated with poor adherence to typical therapies used to treat patients with secondary hyperparathyroidism? Please provide your answers in the boxes below.

| **Therapy** | **Factors associated with poor adherence** |
| --- | --- |
| **Phosphate binders** |  |
| **Calcium supplements** |  |
| **Vitamin D/calcitriol** |  |
| **Calcimimetics** |  |
| **Other (please specify)** |  |

1. In your practice, what resources are available to a typical patient with secondary hyperparathyroidism to aid in their management of secondary hyperparathyroidism? Please explain below:

| Resource | Mark if available | Mark if used | Please explain here |
| --- | --- | --- | --- |
| Dietary counseling |  |  |  |
| Access to a specialist dietician |  |  |  |
| Informational materials (diet/lifestyle) |  |  |  |
| Diaries |  |  |  |
| Informational materials (medications) |  |  |  |
| Nursing follow up |  |  |  |
| Website information |  |  |  |
| Psychological support/counseling |  |  |  |
| Virtual medical/counseling sessions |  |  |  |
| Other |  |  |  |

## **2. Patient Questionnaire.**

**Secondary HYERPARATHYROIDISM TREATMENT CONSENSUS**

**DELPHI PANEL–Patient Questionnaire Outline**

**DELPHI PANEL—Guide to completing the patient questionnaire**

**Introduction**

Thank you for agreeing to participate in the Delphi process, aimed at capturing the current management of secondary hyperparathyroidism. This guide is designed to help you answer the patient questionnaire.

Secondary hyperparathyroidism is a common complication of chronic kidney disease (CKD) that leads to considerable health issues. Kidney Disease: Improving Global Outcomes (KDIGO), consisting of an international panel of experts, has made recommendations to guide the treatment of adult patients with CKD and secondary hyperparathyroidism.^1^ The goal of the Delphi research is to find out how well the real world care of patients matches the guidelines.

Patient care is complex and treatment plans can vary considerably between patients and/or may change over time. Please consider the treatments that **you are currently prescribed** for secondary hyperparathyroidism and **your own treatment history** when answering the majority of these questions. If you feel that many patients may share a particular experience or opinion that is similar to or different from your own, please feel free to provide those details in the comments section in addition to providing your own opinion or experience.

We would like you to provide your opinion based upon **your own experience**. Your experience may not involve all of the management options discussed in the questionnaire. If you do not feel you can offer an opinion regarding specific management options, simply note this in the comment section provided.

In some cases, questions may be preceded by treatment recommendations, guidelines, or opinions from experts in the field (see the white boxes in the sample, pages 2–3) to provide you with background information for the specific questions. These are for your information only and should not influence your responses. Your answers are confidential and will only be included as anonymous patient participant feedback in the study report.

Thank you for your participation in this research.

**DELPHI PATIENT Questionnaire**

**Relationship with Doctor and Clinical Staff**

1. Does your kidney doctor provide you with sufficient information about your secondary hyperparathyroidism and its associated consequences (high parathyroid hormone levels, abnormal calcium levels, high phosphate levels, poor bone health, etc.)? Please provide your answer in the boxes below.

| Yes | No | Please provide any additional comments here |
| --- | --- | --- |
|  |  |  |

1. Are you involved in decisions regarding treatment for your secondary hyperparathyroidism? Please provide your answer in the boxes below.

| Yes | No | Not sure | Please provide any additional comments here |
| --- | --- | --- | --- |
|  |  |  |  |

1. Do you think your kidney doctor listens to your opinion when making treatment decisions for your hyperparathyroidism? Please provide your answer in the boxes below.

| Yes | No | Not sure | Please provide any additional comments here |
| --- | --- | --- | --- |
|  |  |  |  |

1. Does your kidney doctor help you set and review goals related to the management of your secondary hyperparathyroidism that are important to you (e.g., reducing doses of certain medication, reducing the number of medications taken, getting parathyroid hormone and/or phosphate levels under control)? Please provide your answer in the boxes below.

| Yes | No | Not sure | Please provide any additional comments here |
| --- | --- | --- | --- |
|  |  |  |  |

**Treatment Facilities and Appointments**

1. Overall, have your kidney care facilities helped educate you about your secondary hyperparathyroidism through interactions with staff or educational materials (e.g., pamphlets, videos, websites)? Please provide your answer in the boxes below.

| Yes | No | Not sure | Please provide any additional comments here |
| --- | --- | --- | --- |
|  |  |  |  |

**Evaluation and Monitoring**

**The stages of CKD are defined by the Glomerular Filtration Rate (GFR)^1^, which is a measurement of how well the kidneys are working.^1^**

| **CKD Stage** | **GFR** | **Description of Kidney Function** |
| --- | --- | --- |
| Grade 1 (G1) | ≥90 | Normal or High |
| Grade 2 (G2) | 60–89 | Mildly Decreased |
| Grade 3a (G3a) | 45–59 | Mildly to Moderately Decreased |
| Grade 3b (G3b) | 30–44 | Moderately to Severely Decreased |
| Grade 4 (G4) | 15–29 | Severely Decreased |
| Grade 5 (G5) | <15 | Kidney Failure |
| Grade 5D (G5D) | <15 | Kidney Failure with Dialysis |

[**KDIGO 2017, Guideline 3.1.1^1^**](https://kdigo.org/wp-content/uploads/2017/02/2017-KDIGO-CKD-MBD-GL-Update.pdf)

Serum levels of calcium, phosphate, parathyroid hormone (PTH), and alkaline phosphatase activity should be monitored beginning in CKD Grade 3a (recommendation strength 1C).

[**KDIGO 2017, 3.1.2^1^**](https://kdigo.org/wp-content/uploads/2017/02/2017-KDIGO-CKD-MBD-GL-Update.pdf)

The frequency of monitoring serum calcium, phosphate, and PTH should be based on the presence and magnitude of abnormalities (eg, high phosphate levels, high PTH levels), and the rate of progression of CKD (Not Graded).

[**KDIGO 2017 4.2.1^1^**](https://kdigo.org/wp-content/uploads/2017/02/2017-KDIGO-CKD-MBD-GL-Update.pdf)

For patients with CKD Grade 3a–Grade 5 not on dialysis, PTH levels should be serially determined starting at stage Grade 3a. KDIGO 2017 changed this from a single value of PTH to serial results for clinical decisions to detect “progressively rising” or “persistently elevated” PTH levels (recommendation strength 2C).

1. At what stage of CKD and/or parathyroid hormone level did your parathyroid hormone levels become a concern for you and/or your kidney doctor?

| CKD stage, estimated Glomerular Filtration Rate (kidney function) | CKD G3a, 45–90 | CKD G3b, 30–44 | CKD G4, 15–29 | CKD G5, <15 | CKD G5D, <15 with dialysis | Not sure | Please provide any additional comments |
| --- | --- | --- | --- | --- | --- | --- | --- |
|  |  |  |  |  |  |  |  |
| Parathyroid hormone, pg/mL | <150 | 150–300 | 300–599 | 600–1000 | ≥1000 | Not sure | **Please provide any additional comments** |
|  |  |  |  |  |  |  |  |

1. How often are your calcium, phosphate, and parathyroid hormone levels tested? Please indicate your answer by marking the appropriate box.

|  | Testing frequency | | | | |
| --- | --- | --- | --- | --- | --- |
| Test | Every 3 months | Every 6 months | Every 6–9 months | Yearly | Not sure |
| Parathyroid hormone |  |  |  |  |  |
| Calcium |  |  |  |  |  |
| Phosphate |  |  |  |  |  |

1. Has the monitoring frequency for calcium, phosphate, or parathyroid hormone levels changed at any point for any reason? Please mark your answer below and explain.

| Yes | No | If yes, please explain here |
| --- | --- | --- |
|  |  |  |

1. Have you ever had any tests to measure your bone health such as bone density scan or bone markers? Please provide your answer in the boxes below.

| Yes | No | Please provide any additional comments here |
| --- | --- | --- |
|  |  |  |

1. How often do you undergo bone tests? Please indicate your answer by marking the appropriate box below.

| Every 6–9 months | Yearly | Other (please specify) | Unsure |
| --- | --- | --- | --- |
|  |  |  |  |

1. Do you undergo any additional tests as part of your treatment plan for secondary hyperparathyroidism? Please mark the other tests that you have taken below and provide any additional thoughts or comments as needed.

| Test | Mark if taken | Please provide any additional comments here |
| --- | --- | --- |
| Vitamin D level |  |  |
| Fibroblast growth factor 23 (FGF23) level |  |  |
| Other–please specify |  |  |

1. Do you know the reason why each of the tests (if any) related to your secondary hyperparathyroidism were ordered? For example, calcium, phosphate, PTH, or bone tests. Please provide your answer in the boxes below.

| Yes | No | Please provide any additional comments here |
| --- | --- | --- |
|  |  |  |

1. Did anyone explain the results of your tests related to your secondary hyperparathyroidism (eg, calcium, phosphate, parathyroid hormone, bone tests) in a way that was easy for you to understand? Please provide your answer in the boxes below.

| Yes | No | Please provide any additional comments here |
| --- | --- | --- |
|  |  |  |

1. Would you mind if you had to be tested more often than you are currently tested? Please provide your answer in the boxes below.

| Yes | Sometimes | No | Not sure | Please provide any additional comments here |
| --- | --- | --- | --- | --- |
|  |  |  |  |  |

1. What factors make frequent testing easier or more difficult? Please provide your answers in the boxes below.

| Easier (I am more likely to agree to increased testing if…) | More difficult (I am less likely to agree to increased testing if…) |
| --- | --- |
|  |  |

1. Do you usually receive tests results for your secondary hyperparathyroidism in an acceptable period of time? Please provide your answer in the boxes below.

| Yes | No | Not sure | Please provide any additional comments here |
| --- | --- | --- | --- |
|  |  |  |  |

**Interventions**

1. When did you begin taking prescription medication for secondary hyperparathyroidism? Please provide your answer in the box below.
2. Did your doctor explain why you needed to begin treatment with prescription medicine for secondary hyperparathyroidism? Please provide your answer in the boxes below.

| Yes | No | Don’t know | Please provide any additional comments here |
| --- | --- | --- | --- |
|  |  |  |  |

1. If your doctor explained why you needed to begin treatment for your secondary hyperparathyroidism, was the decision based on any laboratory (eg, blood or urine tests) findings? Please provide your answer in the boxes below.

| Yes | No | Don’t know | Please provide any additional comments here |
| --- | --- | --- | --- |
|  |  |  |  |

1. If the decision to begin treatment was based on laboratory findings, what findings were discussed with you by your kidney doctor? Please mark your answer in the box below choosing all that apply. Please provide any additional comments you feel are necessary.

| Laboratory Test | Mark if discussed | Please provide any additional comments here |
| --- | --- | --- |
| Parathyroid hormone |  |  |
| Phosphate |  |  |
| Calcium |  |  |
| Other (please specify) |  |  |

1. What prescription medications are you currently taking for secondary hyperparathyroidism? Please list any prescription medications **prescribed by your kidney doctor** that you are **currently** taking in the box below.
2. What over-the-counter medications or supplements are you currently taking for secondary hyperparathyroidism? Please list any over-the-counter medications or supplements you are **currently** taking in the box below.
3. Do you think the treatments you are receiving for secondary hyperparathyroidism were specifically chosen to meet your individual medical, personal, and financial situation? Please provide your answer in the boxes below.

| Yes | No | Not sure | Please provide any additional comments here |
| --- | --- | --- | --- |
|  |  |  |  |

[**National Kidney Foundation - Kidney Disease Outcomes Quality Institute commentary^2^**](https://www.sciencedirect.com/science/article/pii/S0272638617308983?via%3Dihub) **on KDIGO 2017, 4.1.8**

Labelling of phosphate content in processed foods is frequently incomplete or inaccurate. Moreover, the consumption of raw, organic, and less processed foods, which typically have lower phosphate content, may be economically prohibitive for many patients as kidney failure disproportionately burdens those living in poverty.

1. How easy is dietary phosphate restriction for you in your management of secondary hyperparathyroidism? Please explain your answer below, and include any specific problems you might face in following dietary changes or restrictions.

| Very easy | Somewhat easy | Neither easy nor difficult | Somewhat difficult | Very difficult | Please provide any additional comments here |
| --- | --- | --- | --- | --- | --- |
|  |  |  |  |  |  |

1. Does your kidney doctor or anyone else involved in your kidney care give you the advice you need on your diet and fluid intake? Please provide your answer in the boxes below.

| Yes | No | Not sure | Please provide any additional comments here |
| --- | --- | --- | --- |
|  |  |  |  |

1. If your kidney doctor or kidney care team provides advice, is it clear and consistent? Please provide your answer in the boxes below.

| Yes | No | Not sure | Please provide any additional comments here |
| --- | --- | --- | --- |
|  |  |  |  |

1. How frequently does cost factor into the treatment choices you and your kidney doctor make in the management of your secondary hyperparathyroidism? Please provide your answer in the boxes below.

| Always | Often | Sometimes | Rarely | Never | Please explain here |
| --- | --- | --- | --- | --- | --- |
|  |  |  |  |  |  |

1. Please rank the following factors in order of importance when deciding on treatment choices for secondary hyperparathyroidism from 1, indicating the most important factor, to 7, indicating the least important factor.

| Convenience (eg, easy to obtain, easy to take, given to you during your normal appointments) | Out-of-pocket cost or co-payment that you pay | Doctor’s opinion | How you take the medication (eg, as an oral tablet or by IV/injection) | Pill burden  (total number of pills taken each day for all medical conditions) | Scientific Evidence | Other (please specify) |
| --- | --- | --- | --- | --- | --- | --- |
|  |  |  |  |  |  |  |

1. Have you refused a suggested therapy for your secondary hyperparathyroidism because of pill burden (ie, because of the number of pills you have to take each day for all medical conditions)?

| Yes, often | Yes, sometimes | Yes, rarely | No, never | Please provide any additional comments here |
| --- | --- | --- | --- | --- |
|  |  |  |  |  |

1. In your experience, what factors made it difficult to take calcimimetics or other medications for secondary hyperparathyroidism as prescribed? Consider factors such as how the drug is taken (oral tablet or injected), the size and number of tablets, taste, time of day/night taken, taken with or without food, needs refrigeration, cost of the treatment to you (out-of-pocket or copayment), side effects, etc. Please provide your answers in the boxes below, commenting on any calcimimetics or other medications that you have used at any time. Example brand and generic names of calcimimetics are provided for your reference.

| **Therapy** | **Factors that make taking the treatment difficult as prescribed** |
| --- | --- |
| **Calcimimetics**  *Examples include:*  Sensipar^®^ (cinacalcet)  Parsabiv^®^ (etelcalcetide) |  |
| **Other (please specify**) |  |

1. In your experience, what other resources can you use to help you manage your secondary hyperparathyroidism? Please mark your answers in the boxes below and explain:

| Resource | Mark if available | Mark if you use | Please explain here |
| --- | --- | --- | --- |
| Dietary counseling |  |  |  |
| Access to a specialist dietician |  |  |  |
| Informational materials (diet/lifestyle) |  |  |  |
| Diaries |  |  |  |
| Informational materials (medications) |  |  |  |
| Nursing follow-up |  |  |  |
| Website information |  |  |  |
| Psychological support/counselling |  |  |  |
| Virtual medical or counselling sessions |  |  |  |
| Other |  |  |  |

1. Have any interventions (eg dietary modification), medications, or resources that you have taken or used for your secondary hyperparathyroidism had an impact on your ability to perform daily activities? In the box below, please provide comments on any medications or interventions that have improved or worsened your ability to perform daily activities.
2. Have any interventions (eg dietary modification), medications, or resources that you have taken or used for your secondary hyperparathyroidism had an impact on the quality of your personal or family life? In the box below, please provide your comments on any medications or interventions you think have changed your quality of life for better or for worse.
3. Are there any aspects of your treatment for secondary hyperparathyroidism that were not covered in this questionnaire? In the box below, please comment on any aspect that you believe is relevant.
